# Demonstrating the benefit of a cellulitis-specific patient reported outcome measure (CELLUPROM^©^) as part of the National Cellulitis Improvement Programme in Wales

**DOI:** 10.1186/s41687-024-00754-4

**Published:** 2024-07-10

**Authors:** Marie Gabe-Walters, Melanie Thomas, Linda Jenkins

**Affiliations:** 1 Lymphoedema Wales Clinical Network, Swansea Bay University Health Board, Swansea, Wales; 2 National Cellulitis Improvement Programme Lead, Lymphoedema Wales Clinical Network, Swansea Bay University Health Board, Swansea, Wales

**Keywords:** Cellulitis, Erysipelas, Patient reported outcome measures, Value-based healthcare

## Abstract

**Purpose:**

Despite a known risk of cellulitis recurrence, the management of the wider impact and risk factors has been neglected. The innovative National Cellulitis Improvement Programme (NCIP) addresses this by providing evidence-based and individualised care to improve patient reported outcomes and reduce the risk of recurrence. The aim of this paper is to examine the longer-term impact of cellulitis and to identify a suitable and clinically relevant Patient Reported Outcome Measure (PROM).

**Methods:**

A review of existing cellulitis-specific PROMs was undertaken, alongside literature detailing the patient-focused impact of cellulitis, to identify a suitable PROM for clinical use. A group of expert therapists and patient representatives (*n* = 14) shared their individual and collective experiences over a series of events to discuss and debate the impact of cellulitis and review available PROMs. CELLUPROM^©^ is introduced with anonymised PROM data and case study information reported to establish the impact of CELLUPROM^©^ within usual NCIP care.

**Results:**

No cellulitis-specific PROMs were identified. Literature focused on the signs and symptoms of an acute episode of cellulitis, with outcome measures primarily used to evidence the impact of an intervention. An enduring physical, social and emotional impact of cellulitis was identified in this study, providing the basis for the new cellulitis-specific PROM (CELLUPROM^©^), which has been implemented with good effect in clinical care.

**Conclusion:**

This study has highlighted the lasting impact of cellulitis. Using CELLUPROM^©^ within the risk-reduction NCIP has helped develop Value-Based Healthcare and support programme evaluation.

**Supplementary Information:**

The online version contains supplementary material available at 10.1186/s41687-024-00754-4.

## Introduction

Cellulitis is an acute bacterial skin infection that, if not recognised and managed appropriately, can lead to emergency hospital admissions and sepsis. In the UK, cellulitis is implicated in over 1% of emergency admissions [1], with infections of the skin and subcutaneous tissues accounting for 1.7% (5,074/292,657) of emergency admissions in Wales [2]. A recent study demonstrated that the annual cost of cellulitis in Wales is over £28 million, with UK National Health Service costs expected to be in excess of £571 million [3]. However, these costs focus on the short-term impact and do not account for wider costs to society and patients. In the short term, cellulitis typically affects the lower limb causing pain, erythema, swelling, malaise, nausea and vomiting, however longer-term impacts are likely given the propensity for recurrence and the implication of risk factors. Lymphoedema is identified as one of the most important risk factors [4–6], with opportunities to reduce this risk with skin care, activity and compression garments [7]. Other well known risk factors that can also be reduced with appropriate education, advice and support include: being overweight or obese; damaged skin (fungal infections, ulcers, wounds) and; chronic venous insufficiency. Estimates of cellulitis recurrence vary with between 10 and 50% of patients experiencing another episode [8–14]. Despite the risk of recurrence, studies have focused on optimising antibiotic treatment in the acute phases, neglecting to target the known risk factors for cellulitis, particularly lymphoedema or skin care issues [14–15], or the longer-term impact for patients.

### The National Cellulitis Improvement Programme

**Fig. 1:**
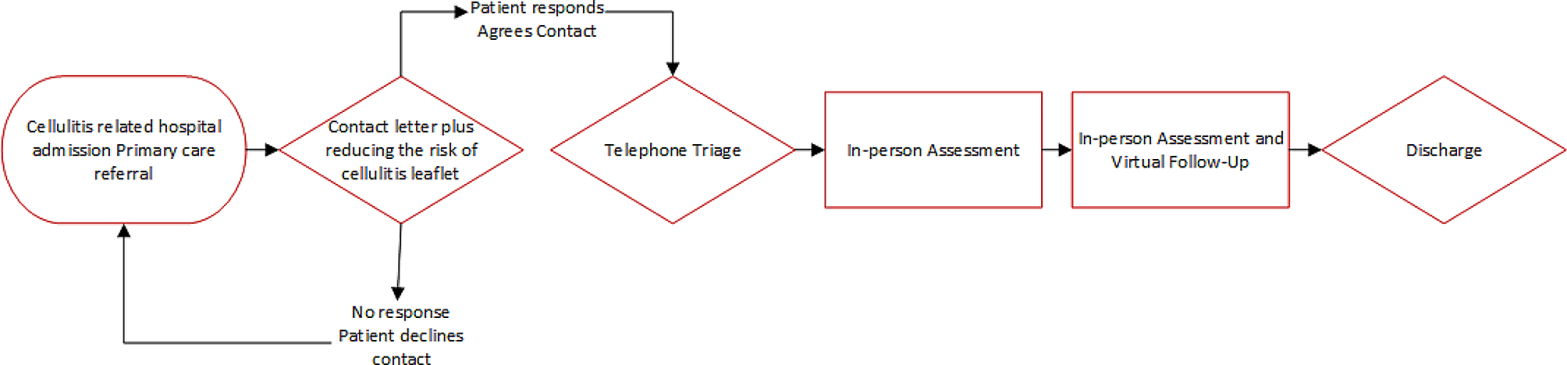
Example National Cellulitis Improvement Programme (NCIP) pathway

A pioneering NHS-based initiative in Wales, known as the National Cellulitis Improvement Programme (NCIP), launched in 2020 under the auspices of Lymphoedema Wales Clinical Network (LWCN). Uniquely, the NCIP provides a cellulitis risk reduction programme to patients (typically over a six month period) [Fig. 1] with a goal to improve their outcomes based on what matters most to them. Supporting patients into the longer-term ‘recovery’ phase provides an opportunity for patients to share their perspective and for therapists to empower evidence-based risk reduction behaviours.

**Fig. 2:**
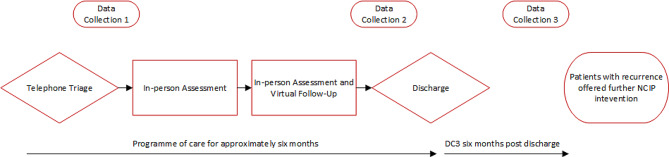
PROM and PREM collection within the NCIP

Many patients report a persisting social, physical or emotional impact of a past cellulitis. Thus, within the ethos of value-based healthcare and to enable evaluation of the NCIP, the systematic collection of patient reported information was embedded in usual care from the outset [Fig. 2]. The EQ5D-5L [16] was collected to help therapists understand the general health status of their patients, however, a limitation was its lack of sensitivity in reporting on the cellulitis-specific impact. In the absence of a validated cellulitis-specific Patient Reported Outcome Measure (PROM), and in part addressing the constraints of the EQ5D-5L, the NCIP developed a cellulitis-specific PROM (CELLUPROM^©^).

The aim of this paper is to report on the journey from conception to inception of the CELLUPROM^©^ in the NCIP. To achieve this aim, this paper will report on the longer-term impact of cellulitis for patients; identify the benefits of establishing a cellulitis-specific PROMs and; report on the impact of CELLUPROM^©^ as part of usual clinical care. This work will support holistic patient care by addressing the gnawing life impact of cellulitis that can persist beyond the acute episode. This paper will then present anonymised routinely collected CELLUPROM^©^ data, alongside case study information. Images (online supplementary file 1–2) have been used with consent from the patient for their use in publication. The full evaluation of the NCIP and validation of CELLUPROM^©^ will also be published in due course with oversight from the local Research and Development team.

## Methods

This paper outlines the work undertaken by the NCIP to establish the need for, and benefit of, routine collection of a cellulitis-specific PROM. There are three complimentary phases to this work including a review of published literature; expert discussion with therapists and patient groups and; an evaluation of anonymous NCIP PROM data, along with case study narratives.

### Phase one

To provide an overview of the current understanding of cellulitis impact, a review of the literature was undertaken using medical subject headings and key word searches (such as cellulitis, quality of life, health-related quality of life and patient reported outcome measures) in electronic databases including CINHAL, Embase, and Medline. The search strategy was developed with oversight from therapists, researchers and a librarian. The titles and abstracts of English language articles published to July 2023 were reviewed by the researcher and their full text retrieved if relevant to the aims of this paper. Studies were excluded if they reported on children (those aged 17 or under) or animal studies. The rigor of existing validated PROMs identified in this search were reviewed using the COSMIN framework [17].

### Phase two

Expert (therapists and patient group’s) views and opinions were garnered to understand the clinical suitability and utility of PROM collection. A series of discussions were undertaken by experts (academics and therapists working in lymphoedema and/or cellulitis *n* = 10), along with volunteer patient representatives (*n* = 4) during 2020. Where appropriate, reviews also occurred independently to expedite the process. During the reviews, there was an opportunity to explore and discuss experiential knowledge and examine the wider impact of cellulitis. Key themes reported by clinician and patient groups were used to appraise the appropriateness, completeness and feasibility of identified PROMs and to identify the items for a cellulitis-specific PROM (CELLUPROM^©^).

### Phase three

Anonymous NCIP CELLUPROM^©^ data are presented with case study information, plus a summary of clinical implementation of PROMs as part of routine care. CELLUPROM^©^ is routinely collected with the EQ5D-5L and a Patient Reported Experience Measure (CELLUPREM^©^) over three data collection (DC) points from one day post triage to six months post discharge [Fig. 2].

## Results

### Phase one: the longer-term impact of cellulitis and its measurement in clinical care

There was a scarcity of literature exploring the wider patient impact of cellulitis beyond the acute illness, such as pyrexia, malaise, pain, nausea or vomiting, erythema and oedema, and no cellulitis-specific PROM was identified in clinical care. Literature predominantly focused on the incidence, diagnosis and treatment of an acute cellulitis. Despite a risk of recurrence in up to half of patients, there was a paucity of studies examining preventative strategies or discussing longer-term patient-focused outcomes. The majority of studies reviewed collected outcome data to examine clinical response or treatment success [18]. A review of published trials, alongside a patient and healthcare professional survey (*n* = 401), identified that a minority of trials (4/42) evaluated Quality of Life (QoL) [19]. A variety of generic measures were identified in these studies, including patient satisfaction with treatment (examining convenience and effectiveness), impression of improvement of cellulitis, the Dermatitis Life Quality Index (DLQI) and the European Quality of Life 5-Dimensions questionnaire (EQ-5D) [13, 19–21]. A smaller number of studies used a lymphoedema-specific PROM [20] with the incidence of post-operative cellulitis reported [22]. Disease specific PROMs (such as dermatology) were identified elsewhere, but none were considered ready to use in clinic care [23] or met the requirements identified by review with therapists and patient groups (phase two). Meanwhile, pain was assessed using a variety of tools including a Likert scale, severity rating, visual analogue scale or specific measures such as McGill pain score or the Brief pain inventory [19]. Once again, the focus of these measurements was to evidence the impact of treatment, rather than improve or understand what it meant for patients living with cellulitis into the longer-term.

A qualitative study using semi-structured group (*n* = 15) and individual (*n* = 9) telephone review of adults admitted for a community-acquired cellulitis concluded that services must be flexible and able to respond to individual need with a greater focus on how patients can reduce their risk of recurrence [24]. In this study, anxiety and dissatisfaction were linked with poor symptom control, negative attitudes and substandard communication. In the recovery phase, a focus was abating the systemic symptoms and returning to normal activities, with provision of information associated with confidence and satisfaction. More recently, as part of international prospective study examining QoL of adults with chronic oedema, 32% (*n* = 347) of patients with lymphoedema received cellulitis advice [25]. Taking stock of these studies, the NCIP answers this call to action and addresses the challenges in accessing specialists [26] or receiving cellulitis-specific advice with the view to improve patient outcomes and reduce the risk of cellulitis recurrence.

### Phase two: expert views on the need for CELLUPROM^©^ as part of the NCIP remit

Expert experiences of having or caring for someone with cellulitis were collated over a series of individual and group discussions; identifying that physical (pain and walking), social (personal care, home life, work/finances, hobbies and holidays) and emotional (body image, intimacy/desirability, anxiety and fear or recurrence) impacts sustained after recovery from an acute cellulitis. For example, patients report challenges in living with discoloured skin that draws attention or questions from those around them. Others were often fearful of having another episode. Taking stock of the multi-domain impact of cellulitis and a dearth of studies focused on cellulitis-specific PROMs, the experts together identified the need for CELLUPROM^©^ as part of usual care.

### The origin of CELLUPROM^©^

Given the overlap and intricate link between cellulitis and lymphoedema, the LYMPROM^©^ [27] provided a framework for experts to evaluate and modify. In particular the group reviewed the relevance, clarity and comprehensiveness of the LYMPROM^©^ for use in cellulitis such as:

- The appropriateness of the response options
- Redundant items that warranted removal
- Missing items that warranted inclusion
- Feasibility and acceptability of use for patients and therapists

**Fig. 3:**
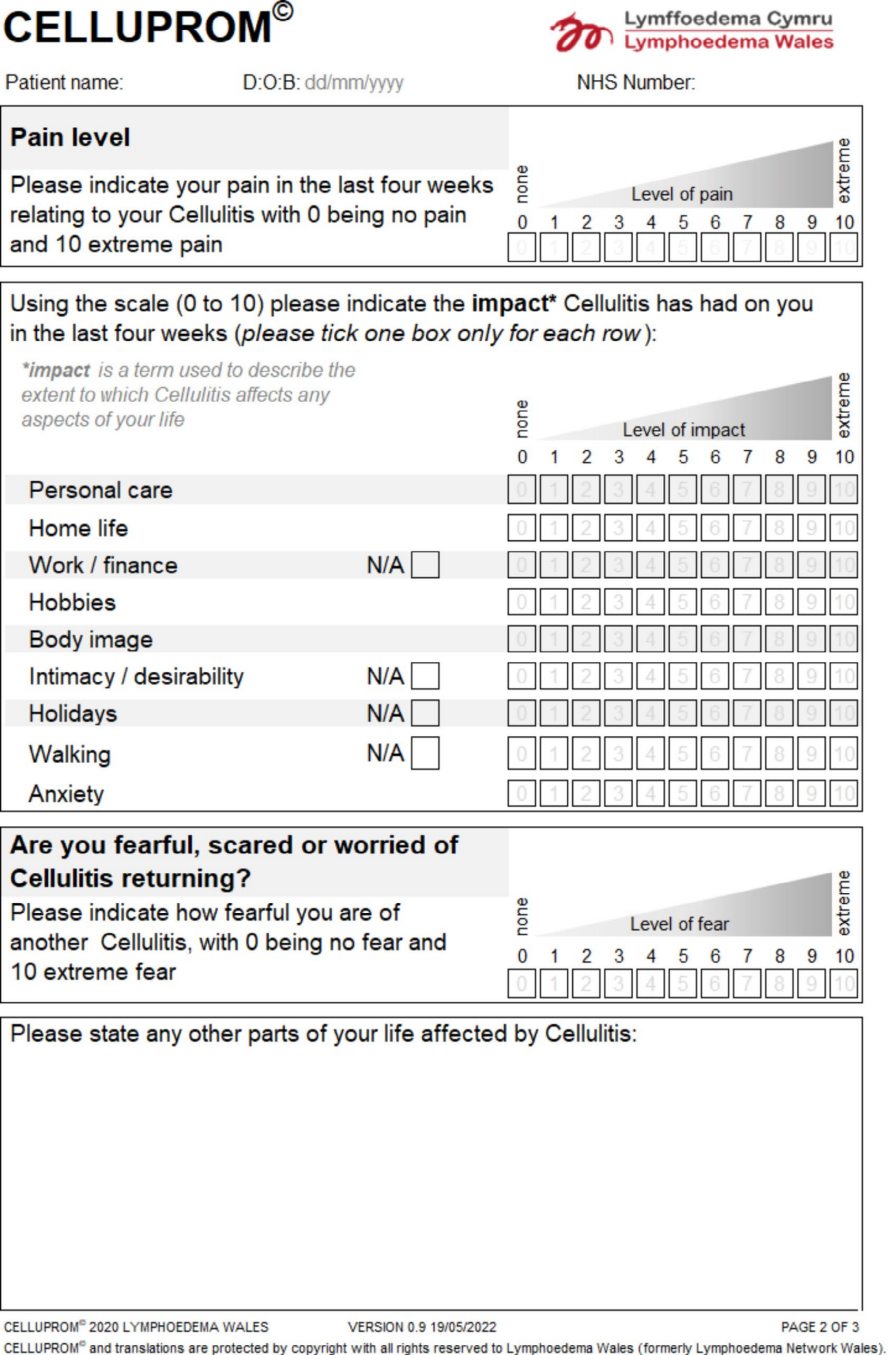
Excerpt of CELLUPROM^©^

The four-week reference period and the eleven-point response scale from zero to 10 (where zero represents no impact and 10 is extreme impact) with ‘not applicable’ options were retained owing to perceived acceptability within clinical care. Next, the group reviewed the relevancy and totality of outcomes for patients with a past or current cellulitis. Firstly, items were reviewed to identify any redundant or irrelevant outcomes for patients with cellulitis (such as heaviness or shopping for clothes/shoes). To ensure a comprehensive coverage of items, the group considered what other relevant outcomes were missing (such as fear of cellulitis recurrence). The 11-item CELLUPROM^©^ was developed with a free text box [Fig. 3] to enable patients to report on any other impact that has not already been covered.

**Fig. 4:**
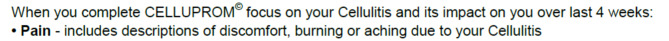
Example of description text on the back page of the paper-based CELLUPROM^©^

**Fig. 5:**
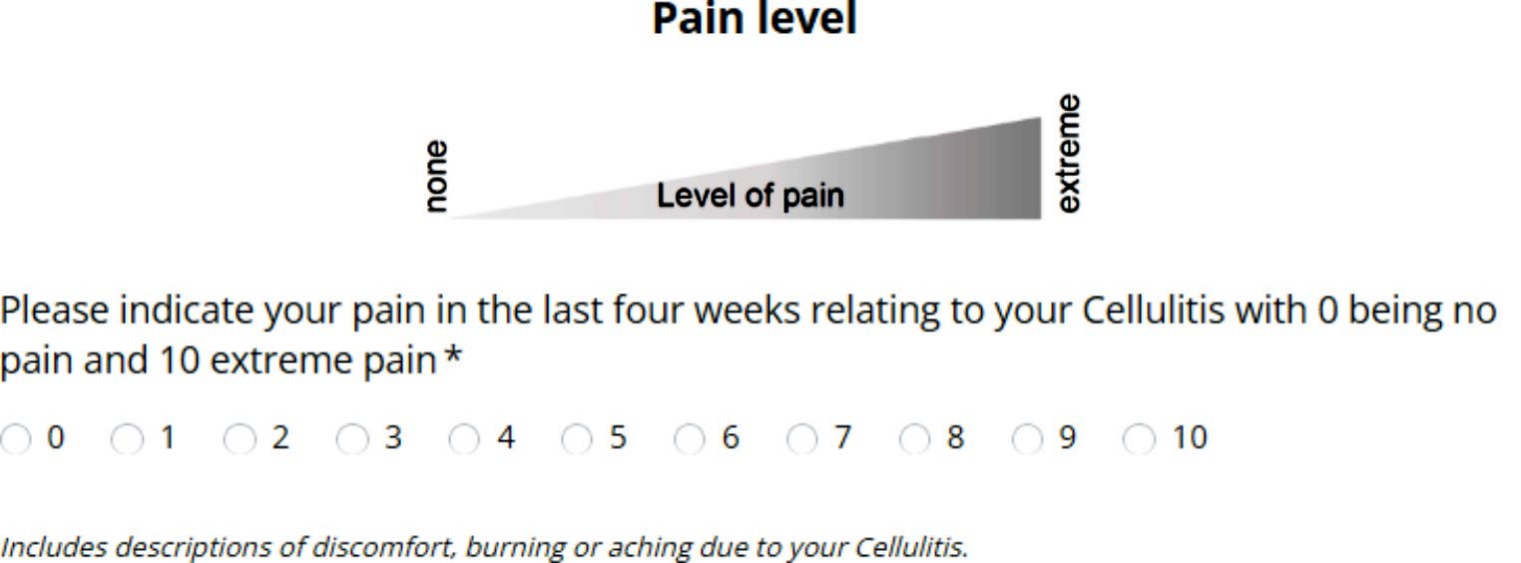
Example of description text on the digital CELLUPROM^©^

CELLUPROM^©^ is available in English and Welsh, with translation following agreed standards [28]. An equivalent digital version of the paper-based CELLUPROM^©^ was created December 2021 following guidelines [29]. In the adaption to digital, a consideration was agreed on the placement of the item descriptions. Chiefly, on the paper-form item descriptions appear overleaf [Fig. 4] whereas on the digital form they appear with each item [Fig. 5]. Ongoing collection of patient reported demographics is also included such as identity, area of body affected by cellulitis, age and number of past cellulitis episodes. There is also an option at the start of the CELLUPROM^©^ to record data collection mode (e.g. paper) and details of the respondent (e.g. proxy).

Proposed work to validate CELLUPROM^©^^,^ following COSMIN guidelines [17], has been ratified by Swansea Bay University Health Board Research and Development as research, which will be published in due course.

### Phase three: CELLUPROM^©^ in clinical care

**Fig. 6:**
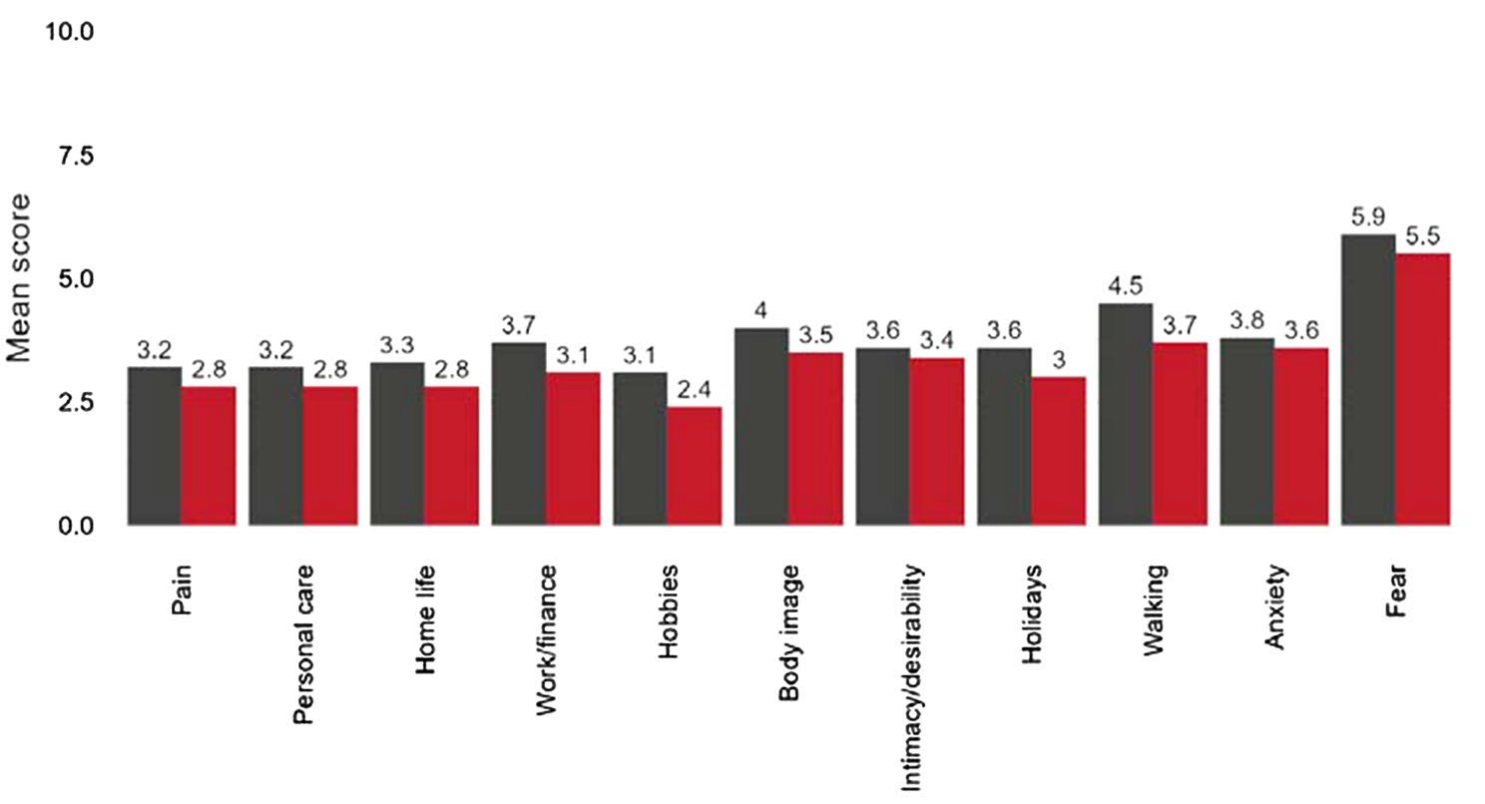
CELUPROM^©^ data showing first and last item scores (*n* = 301)

Using a third-party online platform that is integrated with the NHS patient management system, CELLUPROM^©^ is automatically shared *via* text / e-mail at each of the three DC points [Fig. 2]. All of the individual items within CELLUPROM^©^ and the EQ5D-5L are mandatory, though patients can of course choose not to complete at all. At the end of CELLUPROM^©^ patients are also asked if they you would like to share their experiences (using CELLUPREM^©^). Patients can opt out of communication from the online platform, but can complete a paper-based CELLUPROM^©^, however this data is not then recorded digitally. Feedback is highlighting an acceptance of the value of PROMs in practice, but for many this has been a new way of collaborating in healthcare. Moreover, clinical documentation was adapted to focus on PROM-led care, with each patient contact used as an opportunity to explain and share the value of PROMs in clinical care. Looking at CELLUPROM^©^ data collected at triage and DC2 (three months post-triage) (*n* = 301) shows the emotional burden of cellulitis beyond the acute episode [Fig. 6]. A higher score reflects an increased impact of cellulitis for the patient. Positively, the CELLUPROM^©^ score improved from triage (61.7, SD 30; median 64, IQR 41) to discharge (58, SD 32.1; median 60, IQR 50.9) within an average interval of 105.9 days (SD 65) median 84 (IQR 7).

Moreover, anecdotal feedback from therapists and patients have supported CELLUPROM^©^ as a succinct and pragmatic communication tool to help plan care and monitor the impact of interventions. Two short case studies [supplementary files 1–2] support the important role of PROMs in informing personalised care that provides value based on what matters most to the patient.

## Discussion

Using an integrated digital platform to collect CELLUPROM^©^ has supported timeous data collection. Many patients are embracing digital PROM collection as part of their usual clinical care, however, for many this is the first time they have completed a PROM; highlighting a need for wider emphasis on PROM-led care across the health system. CELLUPROM^©^ has supported PROM-led care within the NCIP. Targeting the complications associated with lymphoedema [30] whilst addressing the lingering experiences of fear and anxiety after ‘recovery’ from the acute phases of an infection. This is important as fear and stress can profoundly impact on QoL and health: exacerbating the symptoms of the disease, increasing inflammation and impairing the immune system [31–34]. The reduced impact of fear, as captured using CELLUPROM^©^, might be explained by patients receiving targeted risk reduction information and information about the potential causes of their cellulitis. The NCIP provides patients with the tools to consider the context of their environment to negotiate their risk of recurrence by promoting psychological well-being and self-efficacy, whilst reducing their fear of recurrence by adopting (or adapting) health behaviours [35–37]. However, PROM data in isolation is not a panacea, but is shown in this paper to illuminate priorities of work at the patient level and more broadly for the NCIP. For example, along with the advent of the newly developed psychology programme within LWCN and updates to clinical documentation (prioritising PROM discussions and documentation), it is likely that these benefits will continue to grow.

This paper has outlined a model of cellulitis risk-reduction care using the CELLUPROM^©^ that is feasible, sustainable and responsive to change. The findings presented provide an impetus for services to consider the longer-term impact of conditions that are at risk of exacerbation, relapse or recurrences. Moving cellulitis care into the realms of risk reduction and patient reported outcomes is enabling the NCIP to target value-based healthcare and afford benefits to patient, services and providers alike: addressing an unmet healthcare need. The validation of CELLUPROM^©^ is already underway, but this is recognised as a current limitation for this paper. Nonetheless, this paper provides a tantalising taster of the remit, capacity and impact of the NCIP, with further publications of the NCIP underway.

### Strengths and limitations

This paper makes prudent and valuable use of a cohort of experts including volunteer patient representatives and therapists across Wales to further our understanding of the patient-focused impact of cellulitis. Owing to the scarcity of published data, caution should be adopted when interpreting the findings of studies cited. However, it is unlikely that the experiences of the experts vastly differ elsewhere in the UK or globally, though this is an important consideration. The psychometric properties of the CELLUPROM^©^ is unknown. It is vital that the data collected using CELLUPROM^©^ are valid and reliable. Using the COSMIN guidelines [17] a series of iterative studies will be undertaken to examine the psychometric properties of CELLUPROM^©^ for adults with a history of cellulitis. This will be published in due course, along with the evaluation of the NCIP. This paper has not directly reported on the EQ5D-5L data, nor has it reported on the identified risk factors and potentially wider determinants of health, which will be examined in future publications.

## Conclusion

The CELLUPROM^©^ data highlight that patients are living in the shadow of cellulitis after treatment, with a fear of recurrence being a chief concern. Using the CELLUPROM^©^ as part of usual care, the NCIP are able to offer an evidence-based and tailored package of care that focuses on key interventions, based on what matters most to the patient. This paper sheds light on the innovative NCIP and garners the opportunity to provide a programme of care that improves outcomes for patients with cellulitis beyond the acute infection, whilst reducing the risk of cellulitis recurrence.

### Electronic supplementary material

Below is the link to the electronic supplementary material.

Supplementary Material 1

Supplementary Material 2

Supplementary Material 3

Supplementary Material 4

Supplementary Material 5

## Data Availability

Not applicable.
