# Supplementary material for: Demonstrating the benefit of a cellulitis-specific patient reported outcome measure (CELLUPROM©) as part of the National Cellulitis Improvement Programme in Wales
Source: J Patient Rep Outcomes. 2024 Jul 10;8:69. doi: 10.1186/s41687-024-00754-4 (PMC11236826; doi:10.1186/s41687-024-00754-4)
Supplement: Supplementary file 1 — Supplementary Material 1 [file 41687_2024_754_MOESM1_ESM.pdf]

CELLUPROM©

Office use only.
PROM: 1 2 HB:

Lymffoedema Cymru
Lymphoedema Wales

Patient name: D:O:B: dd/mm/yyyy NHS Number:

About the patient **Date you completed this:** dd/mm/yyyy

Unless instructed, only tick one answer for each question

Is this the first time you have completed a CELLUPROM© form?

Yes ☐ *If Yes, go to 'Who is completing this?'*

No ☐ *If No, go to next question*

Do you currently have or have you had Cellulitis since you were last seen?

Yes ☐ *Please ring to arrange an appointment with the Cellulitis Service. Contact details can be found at the end of the form. Please continue to complete the rest of the questions.*

No ☐

Who is completing this?

Unless instructed, only tick one answer for each question

Are you completing this for someone else?

Yes ☐ *If Yes, go to next question* No ☐ *If No, go to 'More information'*

What is your relationship to the patient?

Spouse / Partner ☐ Parent / Guardian ☐ Other Family Member ☐

Friend ☐ Social-care professional ☐ Health-care Professional ☐

Other ☐

Why is your assistance needed? *Please select all that apply*

The patient has a physical impairment ☐ Other ☐

The patient has a mental / cognitive impairment ☐

The patient does not speak English or Welsh ☐

Remote collection (e.g. telephone completion) ☐

If you are helping someone to complete this self-assessment, please ensure that the information and opinions given are that of the patient and not your own.

More information. *Unless instructed, only tick one answer for each question*

How old are you? *Please specify using the ranges below:*

18-24 ☐ 25-34 ☐ 35-44 ☐ 45-54 ☐ 55-64 ☐ 65-74 ☐

75-84 ☐ 85+ ☐

How do you identify?

Female ☐ Male ☐ Other ☐ Prefer not to say ☐

Where on your body have you had Cellulitis? *Please select all that apply.*

Upper limb (arm / hand) ☐ Lower limb (leg / foot) ☐

Midline (head, neck, trunk) ☐

How many times have you had Cellulitis in the past?

0 ☐ 1 ☐ 2 ☐ 3 ☐ 4 ☐ 5 ☐ 6 or more ☐

CELLUPROM© and translations are protected by copyright with all rights reserved to Lymphoedema Wales (formerly Lymphoedema Network Wales).

CELLUPROM©

Lymffoedema Cymru
Lymphoedema Wales

Patient name: D:O:B: dd/mm/yyyy NHS Number:

Pain level

Please indicate your pain in the last four weeks relating to your Cellulitis with 0 being no pain and 10 extreme pain

Level of pain (none – extreme)

0	1	2	3	4	5	6	7	8	9	10
0	1	2	3	4	5	6	7	8	9	10

Using the scale (0 to 10) please indicate the **impact*** Cellulitis has had on you in the last four weeks (*please tick one box only for each row*):

****impact* is a term used to describe the extent to which Cellulitis affects any aspects of your life*

Level of impact (none – extreme)

		0	1	2	3	4	5	6	7	8	9	10
Personal care		0	1	2	3	4	5	6	7	8	9	10
Home life		0	1	2	3	4	5	6	7	8	9	10
Work / finance	N/A ☐	0	1	2	3	4	5	6	7	8	9	10
Hobbies		0	1	2	3	4	5	6	7	8	9	10
Body image		0	1	2	3	4	5	6	7	8	9	10
Intimacy / desirability	N/A ☐	0	1	2	3	4	5	6	7	8	9	10
Holidays	N/A ☐	0	1	2	3	4	5	6	7	8	9	10
Walking	N/A ☐	0	1	2	3	4	5	6	7	8	9	10
Anxiety		0	1	2	3	4	5	6	7	8	9	10

Are you fearful, scared or worried of Cellulitis returning?

Please indicate how fearful you are of another Cellulitis, with 0 being no fear and 10 extreme fear

Level of fear (none – extreme)

0	1	2	3	4	5	6	7	8	9	10
0	1	2	3	4	5	6	7	8	9	10

Please state any other parts of your life affected by Cellulitis:

CELLUPROM© and translations are protected by copyright with all rights reserved to Lymphoedema Wales (formerly Lymphoedema Network Wales).

CELLUPROM©

Lymffoedema Cymru
Lymphoedema Wales

CELLUPROM© is a Cellulitis Patient Reported Outcome Measure tool to use as part of your clinical assessment.

Cellulitis is a skin infection and can affect any area of your body. Cellulitis is caused when a break in the skin allows bacteria to cause an infection. Cellulitis is not contagious and rarely affects more than one area of your body at the same time. The CELLUPROM© can only be completed if you have or have had Cellulitis.

CELLUPROM© will gather information on things that matter to you and will also support us in monitoring your progress. You will see that the severity of impact is scored as 0 to 10, with 0 being no impact to 10 being extreme impact.

The information you provide is based on how you have felt over the last four weeks. We want to understand if Cellulitis has an impact on your day to day life.

When you complete CELLUPROM© focus on your Cellulitis and its impact on you over last 4 weeks:

- **Pain** - includes descriptions of discomfort, burning or aching due to your Cellulitis
- **Personal care** - includes washing / dressing / looking after yourself
- **Home life** - includes housework / cooking / social life / family life / driving / caring for pets
- **Work / finances** - includes paid & voluntary work as well as activities such as caring for grandchildren or others. Please tick N/A if you are not currently working or do not wish to answer this question
- **Hobbies** - has your Cellulitis impacted types of hobbies such as sports, sewing, reading, gardening, swimming etc?
- **Body image** – Has Cellulitis affected the way you feel about your body?
- **Intimacy / desirability** – Has Cellulitis affected your intimate relationships? Please tick N/A if you do not want to answer this question
- **Holidays** - have you changed holiday plans / destinations due to your past Cellulitis? This includes short breaks and day trips. Please tick N/A if you do not take holidays
- **Walking** – Has Cellulitis affected your balance, how far you can walk, or your normal day to day mobility? Please tick N/A if this is not relevant to you.
- **Anxiety levels** - does Cellulitis make your feel anxious? You can think of anxiety as a feeling that includes being nervous or on edge. It tends to more than a passing worry or fear. You might find it hard to stop worrying or find that you worry ***excessively*** about what might happen if you had another episode of Cellulitis. It may also affect you physically and can affect activities of daily living.
- **Are you fearful, scared or worried of Cellulitis returning?** How scared or worried are you of having another episode of Cellulitis?

We hope this information has been useful in enabling you to complete your own CELLUPROM©.

Contact Details for the Cellulitis Improvement Team:

Cellulitis Improvement Team
Lymphoedema Network Wales
Email:
Tel:

CELLUPROM© and translations are protected by copyright with all rights reserved to Lymphoedema Wales (formerly Lymphoedema Network Wales).
