# Supplementary figures and images for: Demonstrating the benefit of a cellulitis-specific patient reported outcome measure (CELLUPROM©) as part of the National Cellulitis Improvement Programme in Wales
Source: J Patient Rep Outcomes. 2024 Jul 10;8:69. doi: 10.1186/s41687-024-00754-4 (PMC11236826; doi:10.1186/s41687-024-00754-4)

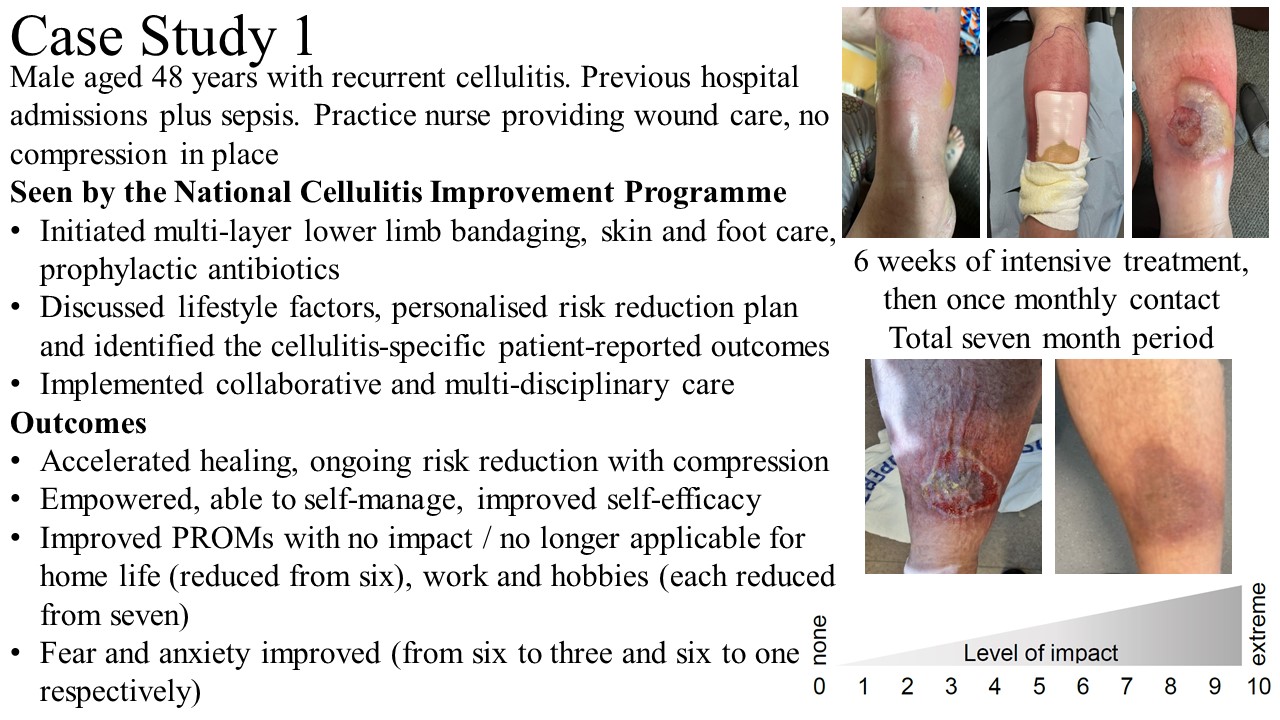

Supplement: Supplementary file 2 — Supplementary Material 2 [file 41687_2024_754_MOESM2_ESM.jpg]

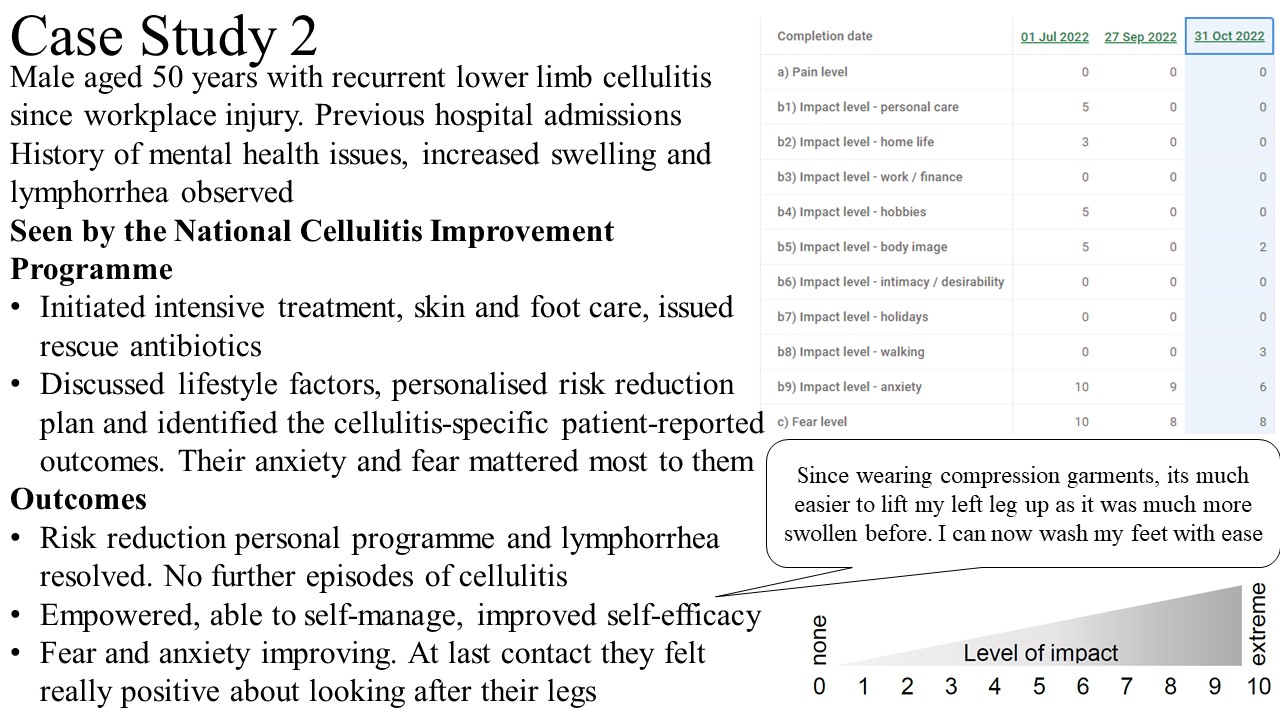

Supplement: Supplementary file 3 — Supplementary Material 3 [file 41687_2024_754_MOESM3_ESM.jpg]

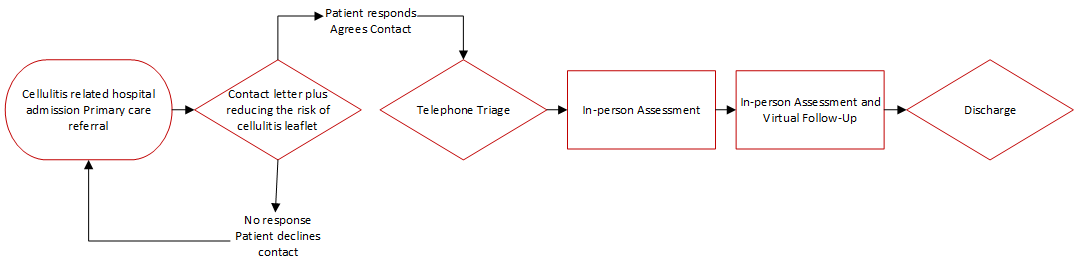

Supplement: Supplementary file 4 — Supplementary Material 4 [file 41687_2024_754_MOESM4_ESM.png]

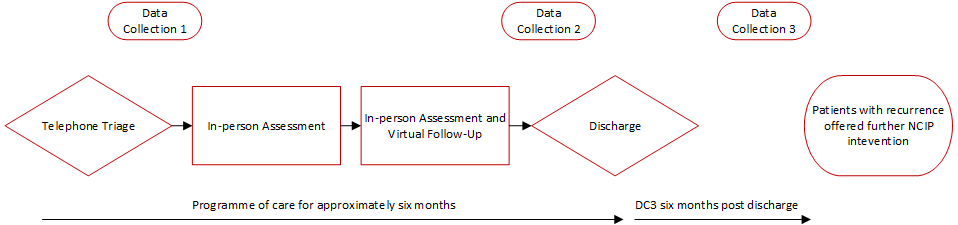

Supplement: Supplementary file 5 — Supplementary Material 5 [file 41687_2024_754_MOESM5_ESM.png]
